# *QuickStats:* Average Number of Stroke* Deaths per Day, by Month and Sex — National Vital Statistics System, United States, 2021

**DOI:** 10.15585/mmwr.mm7249a7

**Published:** 2023-12-08

**Figure:**
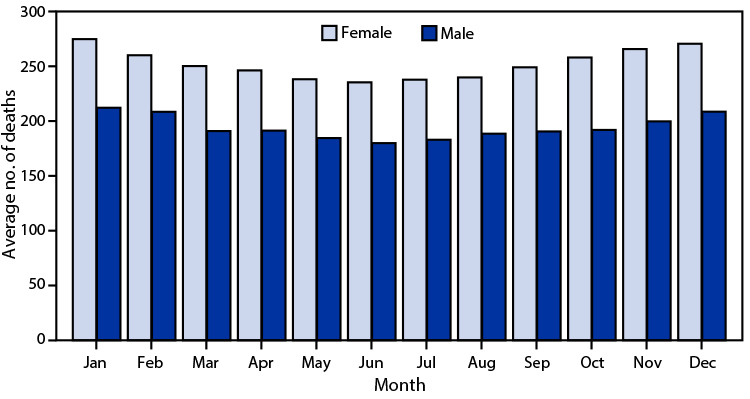
In 2021, the average number of stroke deaths per day was highest in January (275 for females and 212 for males) and then declined to a monthly low in June (235 for females and 180 for males). Beginning in July, the average number of stroke deaths per day increased for each successive month through the end of the year among both males and females, with the average number of stroke deaths higher among females than males for every month.

**Source:** National Center for Health Statistics, National Vital Statistics System, Mortality Statistics, 2001–2021. http://www.cdc.gov/nchs/nvss/deaths.htm

For more information on this topic, CDC recommends the following link: https://www.cdc.gov/stroke/

